# Fatal *Streptococcus pseudoporcinus* disseminated infection in decompensated liver cirrhosis: a case report

**DOI:** 10.1186/s13256-021-02832-3

**Published:** 2021-05-16

**Authors:** George D. Liatsos, Athanasia Tsiriga, Spyridon P. Dourakis

**Affiliations:** 1 Department of Internal Medicine, “Hippokration” General Hospital, 114 Vass. Sophia’s Ave., 115 27 Athens, Greece; 2 Department of Microbiology, “Hippokration” General Hospital, Athens, Greece; 3 2nd Department of Internal Medicine and Research Laboratory, Medical School, National and Kapodistrian University of Athens, “Hippokration” General Hospital, Athens, Greece

**Keywords:** *Streptococcus pseudoporcinus*, Group B streptococci, Spontaneous bacterial peritonitis, Fatal infection, Multidrug-resistant strain

## Abstract

**Background:**

*Streptococcus pseudoporcinus* (*S. pseudoporcinus*) was first identified in 2006. It cross-reacts with Lancefield group B antigen agglutination reagents and has been misidentified as *S. agalactiae*. Sites of *S. pseudoporcinus* isolation include the female genitourinary tract, urine, wounds, and dairy products. The prevalence of vaginal colonization is reportedly between 1 and 5.4%. Two uneventful cases of soft tissue infection caused by *S. pseudoporcinus* were reported in the past. However, since late 2019, six cases of invasive *S. pseudoporcinus* infections have emerged in the literature, one of which was fatal.

**Case presentation:**

We describe a fatal case of a Caucasian male with spontaneous bacterial peritonitis associated with bacteremia due to a multidrug-resistant *S. pseudoporcinus* strain in a patient with decompensated liver cirrhosis. Despite the patient’s good general condition and stable blood test results when he had visited the outpatient clinic for large-volume paracentesis a few days before admission, this time he presented to the emergency department with a rapidly worsening clinical condition and with laboratory features consistent with multiple-organ dysfunction syndrome, and succumbed within a short period.

**Conclusions:**

Contrary to what was thought until recently, multidrug-resistant *S. pseudoporcinus* may cause invasive, disseminated, fatal disease in humans. According to current limited data, vancomycin, linezolid, daptomycin, levofloxacin, clindamycin, and tetracycline seem to be the most effective antimicrobial agents against multidrug-resistant strains, and should be the empirical choice in cases of disseminated *S. pseudoporcinus* infection until laboratory antimicrobial susceptibility results are available. Improvements and new approaches for bacterial identification in routine clinical microbiology laboratories may reveal the real spectrum of *S. pseudoporcinus* infections in humans, which is currently believed to be underestimated. *SS. pseudoporcinus* could emerge as a serious medical problem in the near future, similar to other β-hemolytic streptococci.

## Background

*Streptococcus pseudoporcinus* (*S. pseudoporcinus*) was first described in 2006 after several human isolates recovered from the female genitourinary tract, phenotypically identified as *S. porcinus*, were finally investigated by 16S ribosomal ribonucleic acid (16S rRNA) gene sequencing and were found to be over 2.1% dissimilar to any other *Streptococcus* species [1]. It has biochemical characteristics similar to those of *S. agalactiae* (group B *Streptococcus*, GBS). It often cross-reacts with Lancefield group B antigen agglutination reagents of standard GBS testing kits, thus raising concerns that *S. pseudoporcinus* has been misidentified as GBS in routine cultures.

Apart from female colonization and infections in pregnant women, *S. pseudoporcinus* has been rarely recovered from urine cultures, wounds, endophthalmitis samples, and dairy products [2, 3]. Two uneventful cases of soft tissue infections in which *S. pseudoporcinus* was identified as the virulent factor were also published [4, 5]. Researchers have speculated that *S. pseudoporcinus* might not be associated with invasive disease to the same extent as *S. agalactiae* infection [3]. We present the case of an invasive, disseminated, fatal infection due to a multidrug-resistant *S. pseudoporcinus* strain. We also review six additional cases of *S. pseudoporcinus* bacteremia that recently emerged in the literature.

## Case presentation

A 56-year-old Caucasian man presented to the emergency department because of progressive abdominal distension with discomfort and decreased urine volume for the past 2 days. His medical history was significant for decompensated alcoholic liver cirrhosis, type 2 diabetes mellitus, and coronary heart disease. He was married with two children, lived in an urban area, and was employed as an accountant. He was a nonsmoker and reported abstinence from alcohol for the past 6 months. His medication included insulin glargine and diuretics (furosemide plus spironolactone). One year before admission, after suffering his first episode of variceal bleeding, propranolol in combination with endoscopic variceal ligation was prescribed at a starting dose of 10 mg twice daily. The patient could not tolerate higher propranolol dosing during titration because of bradycardia. Ten months before admission, he was started on prophylaxis with ciprofloxacin 500 mg daily after a spontaneous bacterial peritonitis (SBP) episode, but 3 months later he discontinued antibiotic prophylaxis on his own. Over the past 3–4 months he had more frequent hospitalizations than in the past for large-volume ascites paracentesis.

**Fig. 1:**
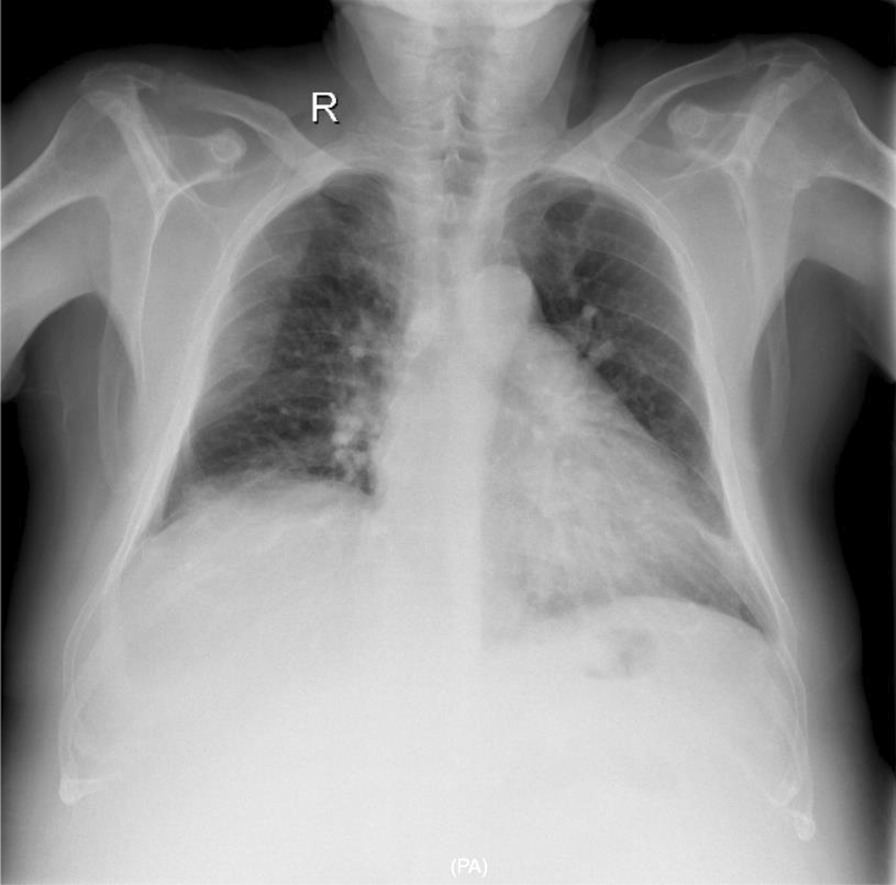
Patient’s chest X-ray on admission

On clinical examination, he was jaundiced, with blood pressure of 90/50 mmHg (baseline systolic blood pressure 120–130 mm/Hg) and heart rate of 72 beats per minute, and he was afebrile (36.7 °C). On skin examination, jaundice and spider angiomata were found on the trunk, face, and upper limbs; gynecomastia was also seen. Neurological examination revealed lethargy, altered mental status, and mild confusion. Neuromuscular impairment was noted including bradykinesia, asterixis (flapping motions of outstretched hands), slurred speech, ataxia, and hyperactive deep tendon reflexes. Focal neurologic deficits were absent. On chest examination, fine crackles were present at the lung bases. The abdominal wall appearance revealed a *caput medusae* due to portal hypertension, while dilated abdominal veins were also seen because of inferior and superior vena cava syndrome. Physical examination also showed a remarkable abdominal distension, a fluid wave, and flank dullness to percussion, with diffuse tenderness and normal bowel sounds. The liver was palpable two fingers below the right costal margin, with firm and nodular consistency; an enlarged left lobe was also palpable. Peripheral edema was more pronounced at the lower extremities. A chest X-ray performed on admission showed mild bilateral infiltrates of the lower lung areas (Fig. 1). Laboratory parameter values showed increased white blood cell (WBC) count and C-reactive protein (CRP), substantial acute kidney injury, and severe deterioration of liver biochemistry compared to a recent previous evaluation (Table 1).

**Table 1:** Patient’s laboratory findings at his previous discharge (performed at our hospital) 10 days before his present admission (first column), and during his current 3-day hospitalization

Laboratory value (normal)	10 Days before admission	Day 1 Admission	Day 2	Day 3
*Blood/serum parameters*				
WBC (< 12,400/μL)	6710	14,070	11,960	12,800
Neut/lymph %	79.8/9.1	93.8/1.9	91/3.3	90.3/2.8
Hct (%)/Hb (g/dL)	27.3/9.1	30.2/9.9	28/9.5	27.2/9.1
PLT (>150,000/μL)	168,000	215,000	239,000	227,000
Urea (< 55)/Creat (< 1.2) mg/dL	58/0.8	134/4.6	163/5.4	174/5.9
Sodium > 136/potassium < 5.1 mmol/L	129/4.4	121/5.5	117/6.5	124/5.6
AST (< 34)/ALT (< 55) U/L	241/161	657/351	2071/1002	4812/1774
LDH (< 220 U/L)	290	758	2321	7683
ALP (< 150)/γ-GT (< 64)U/L	148/171	160/172	154/147	233/132
TBIL (< 1.2)/DBIL (mg/dL)	10.48/6.92	13.5/9.38	16.9/11.3	19.8/13.2
TP (> 6.4)/Alb (> 3.5) g/dL	6.9/3.3	7.3/3.3	6.8/3.1	ND
PT (< 14 sec)/INR	14.6/1.3	20/1.8	21.1/2.0	40.6/4.1
CRP (< 5 mg/L)	37.3	118.7	134.4	132.2
*Blood gas analysis*				
pH/HCO_3_mmol/L	7.43/22.1	7.35/16.7	7.33/15.0	7.02/8.1
FiO_2_/SatO_2_%	0.21/97	0.21/96	0.21/98	0.50/98.5
PO_2_/PCO_2_mmHg	89/30	83/25	76/22	94/31
Lactate acid (< 1.8 mmol/L)	1.0	4.0	4.1	9.4
*Ascitic fluid*				
WBC count/μL	80	5920	ND	3680
Neut/lymph%	ND	60/30		80/10
Glu/LDH	130/<90	85/96		ND
TP/Alb g/dL	1.2/0.6	1.9/1.0		ND

*WBC* white blood cells, *Neut/lymph* neutrophil-to-lymphocyte ratio, *Hct* hematocrit, *Hb* hemoglobin, *PLT* platelets, *Creat* creatinine, *AST* aspartate transaminase, *ALT* alanine aminotransferase, *LDH* lactate dehydrogenase, *ALP* alkaline phosphatase, *γ-GT* γ-glutamyltransferase, *TBIL* total bilirubin, *DBIL* direct bilirubin, *TP* total proteins, *PT* prothrombin time, *CRP* C-reactive protein,* FiO*_*2*_ fraction of inspired oxygen, *SatO*_*2*_ oxygen saturation, *PO*_*2*_ partial pressure of oxygen *PCO*_*2*_ partial pressure of carbon dioxide, *Glu* glutamate, *LDH* lactate dehydrogenase, *Alb* albumin, *ND* not done

The patient was evaluated as having sepsis; he was started on intravenous administration of meropenem (1 g three times daily) and daptomycin (350 mg daily) and hydrocortisone 100 mg four times daily and was admitted to the hospital. On day 2, renal function deteriorated further and he was started on terlipressin 3 mg infused over 24 hours in combination with intravenous albumin (1 g/kg on day 1, and then 20 to 40 g/day thereafter) for a possible type 1 hepatorenal syndrome. Later on the same day, he was subjected to a course of hemodialysis due to hyperkalemia (6.5 mmol/L), anuria, and increased central venous pressure (24 cm/H_2_O). On day 3, the patient developed severe metabolic acidosis, his liver failure worsened, and he subsequently fell into a coma. Within the context of multiple-organ dysfunction syndrome (MODS), the patient was intubated, but he later died within just a short period after admission to the hospital. No postmortem examination was performed, as the cascade of causes of the patient’s death was documented. The immediate cause of death was MODS as a consequence of sepsis subsequent to bacteremia due to SBP caused by *S. pseudoporcinus*, which was cultured from the patient’s blood and ascitic fluid (see below). The underlying cause triggering the events resulting in death was decompensated liver cirrhosis.

On admission, a pair of blood and ascitic fluid cultures (pair of both aerobic and anaerobic bottles) was obtained and incubated in a BACTEC 9240 automated system (Becton Dickinson and Company, Franklin Lakes, NJ, USA) for a total of 5 days. The cultures (both blood and anaerobic ascitic fluid) were positive after 3 days of incubation. Ascitic fluid gram stain revealed small gram-positive cocci arranged in chains which grew on both 5% sheep blood and chocolate agar plates after incubation for 24 hours under 5% carbon dioxide conditions. The isolate was catalase-negative. The GP ID card of the VITEK 2 system (bioMerieux, Marcy L’ Etoile, France) identified the isolate as *S. pseudoporcinus*, with excellent identification. Susceptibility testing was performed using the disk diffusion method. Minimum inhibitory concentration (MIC) was determined using the VITEK 2 AST-ST01 card and MIC Test Strips (Liofilchem, Roseto Degli Abruzzi, Italy) (Table 2). Both methods (disk diffusion method and MIC Test Strips) were performed on Mueller-Hinton agar plates supplemented with 5% horse blood and incubated under 5% carbon dioxide conditions for 24 hours.

**Table 2:** Drug susceptibility results of *Streptococcus pseudoporcinus* strain recovered from patient’s blood and ascitic fluid cultures

Antimicrobial agent	Susceptibility (MIC μg/mL)
Ampicillin	R (8)
Amoxicillin clavulanate	R
Penicillin G	R (≥ 8)
Cefuroxime	R
Cefotaxime	R
Ceftriaxone	R (4)
Meropenem	R
Vancomycin	S (0.5)
Daptomycin	S (0.19)
Linezolid	S (≤ 2)
Erythromycin	R
Clindamycin	S (≤ 0.25)
Levofloxacin	S (0.5)
Tetracycline	S (≤ 0.25)
Rifampicin	S
Chloramphenicol	S

*MIC* minimum inhibitory concentration, *R* resistant, *S* susceptible

## Discussion

We report a patient with liver cirrhosis who presented with spontaneous bacterial peritonitis and sepsis, hepatic encephalopathy, type 1 hepatorenal syndrome resulting in metabolic acidosis, acute liver failure, and complications from MODS. Ascitic fluid and blood cultures grew *S. pseudoporcinus*, an emerging, multidrug-resistant pathogen (resistance to penicillin, to third-generation cephalosporins, and even to carbapenems) which has been previously linked to adverse obstetric outcomes and has been widely misidentified as GBS. This is the second case in the literature (PubMed) reporting on a severe, invasive disseminated *S. pseudoporcinus* infection with an untoward outcome, and the first case involving a patient with liver cirrhosis. *S. pseudoporcinus* should be identified as early as possible in such cases, and administration of empirical antibiotics should provide wide coverage against common microorganisms as well as against this important, potentially life-threatening streptococcus.

*S. pseudoporcinus* has biochemical characteristics similar to *S. agalactiae*, and its isolates may cross-react with several GBS antigen agglutination kits, causing it to be misidentified as GBS in routine screening cultures. *S. pseudoporcinus* is a facultative, non-motile, gram-positive coccus arranged in short chains, which produces smooth, round-to-oval, beta-hemolytic colonies on blood agar [3, 6]. In contrast to *S. agalactiae*, which displays a narrow zone of beta-hemolysis, *S. pseudoporcinus* exhibits a wide zone. Like *S. agalactiae*, *S. pseudoporcinus* produces large colonies after 24 hours of incubation. Its catalase and benzidine tests are negative [1]; it is Christie-Atkins-Munch-Peterson (CAMP) factor-positive and bacitracin-resistant [6]. *S. agalactiae* is esculin hydrolysis-negative and hippurate hydrolysis-positive, and does not ferment sorbitol or mannitol, while the opposite is true for *S. pseudoporcinus* [1, 6].

*S. pseudoporcinus* pathogenesis has been linked to adverse obstetric outcomes such as chorioamnionitis and preterm delivery [6]. It is suggested that an ascending *S. pseudoporcinus* infection may trigger an inflammation cascade leading to cervical insufficiency or premature rupture of membranes. Other sites of *S. pseudoporcinus* isolation are wounds, urine, placenta, and dairy products. In the Centers for Disease Control and Prevention (CDC) *Streptococcus* strain collection, scientists evaluated 97 animal, human, and dairy *S. porcinus* or *S. pseudoporcinus* isolates. Seventy-two human and six dairy isolates were identified as *S. pseudoporcinus* [2]. Three-quarters of the specimens were recovered from the genitourinary tract. The rest related to wound infections in male patients. While blood was listed as the source of five isolates, *S. pseudoporcinus* invasive disease was not identified in another CDC *Streptococcus* Laboratory population-based study of invasive disease due to beta-hemolytic streptococci, thus making the existence of those five *S. pseudoporcinus* blood isolates questionable [2]. Nevertheless, according to recently emerging cases of invasive *S. pseudoporcinus* infections in the literature [11–15], the sites of *S. pseudoporcinus* colonization that lead to subsequent bacteremia were presumed to be the gastrointestinal tract and the oropharynx. Regarding *S. porcinus*, it was first isolated from swine in 1937 and was formally described in 1985. The main reservoir is swine, but it has also been isolated from other animals including cattle, sheep, guinea pigs, rabbits, and dogs.

In a study conducted to evaluate screening cultures recovered from vaginal and rectal swabs in pregnant women originating from the Caribbean and sub-Saharan Africa, 15 isolates were identified as *S. pseudoporcinus* (14 from rectovaginal and 1 from urine cultures) [6]. The population prevalence of *S. pseudoporcinus* colonization was determined to be 5.4% in vaginal and rectal swab samples obtained from sexually active, non-pregnant women of reproductive age, all of which cross-reacted with commercially available GBS serogrouping kits [7]. In another study, 717 consecutive *S. agalactiae* screening cultures were received from pregnant women. Of those, 260 (36.3%) that were beta-hemolytic and group B antigen-positive were subsequently subjected to matrix-assisted laser desorption/ionization–time of flight mass spectrometry (MALDI-TOF MS). *S. agalactiae* was confirmed in 248 (34.6%) samples, while six were identified as *S. halichoeri*, and another six as *S. pseudoporcinus*. Researchers mentioned that without MALDI-TOF MS, those 12 specimens would have been falsely identified as *S. agalactiae* [8]. In a large retrospective cohort study of 3704 pregnant women with cultures screened for GBS, the authors concluded that *S. pseudoporcinus* colonization occurred in 1.6% of all pregnancies, whereas a total of 2.5% of all GBS-positive results by agglutination assay were false-positive, instead reflecting *S. pseudoporcinus* colonization [9]. In another prospective observational study among 3276 screening cultures that were collected, 32 isolates (1%) of *S. pseudoporcinus* (25 isolates from pregnant women and 7 from non-pregnant women) and 604 isolates (18.4%) of *S. agalactiae* were identified by MALDI-TOF MS [3]. The identified risk factors for acquisition of *S. pseudoporcinus* colonization resulting collectively from all those studies are shown in Table 3.

**Table 3:** Risk factors for *Streptococcus pseudoporcinus* acquisition and subsequent vaginal-rectal colonization [1–10]

*Risk factors*
African American, Black race, Jamaican, sub-Saharan
Reproductive age
Recent *Trichomonas vaginalis* infection
Primary or recurrent genital herpes
Bacterial vaginosis by Nugent criteria
Two or more sexual partners since the last health clinic visit
Diabetes
Obesity (BMI > 35)
Tobacco use
History or current sexually transmitted or urinary tract infection

*BMI* body mass index

Our patient suffered from a disseminated *S. pseudoporcinus* infection comprising spontaneous bacterial peritonitis (SBP) with bacteremia that was complicated by acute liver failure, type 1 hepatorenal syndrome, stage III hepatic encephalopathy, hypotension, and lactic acidosis. All of these manifestations were attributed to end-organ hypoperfusion (MODS) due to sepsis. *S. pseudoporcinus* in our case could have been acquired by the consumption of contaminated dairy products, since the pathogen has been previously recovered from them [10], with subsequent colonization of the gastrointestinal tract. Colonization of the gut lumen could traverse the intestinal wall and colonize mesenteric lymph nodes (bacterial translocation). Bacterial peritonitis occurs if the lymphatic vessel carrying the contaminated lymph ruptures due to portal hypertension or if the pathogen moves from the mesenteric lymphatic system to the systemic circulation (bacteremia). The bacterial isolate was multidrug-resistant. We administered meropenem, to which it was resistant, and daptomycin, with an MIC of 0.19. The latter was preferred to vancomycin because of the patient’s worsening kidney failure. A second diagnostic paracentesis performed after 48 hours of treatment showed a 40% decrease in ascitic fluid white blood cell (WBC) count and negative cultures. However, the patient at presentation was already severely ill with MODS, and such patients are at very high risk of mortality even with the best supportive treatment.

## Conclusions

*S. pseudoporcinus* is an emerging pathogen that colonizes the female genitourinary tract, with a population prevalence ranging between 1 and 5.4%, and is associated with adverse obstetric outcomes. A few isolates have been recovered from urine cultures and wounds [2, 3] and one from an endophthalmitis sample [10]. In two uneventful cases, *S. pseudoporcinus* was identified as the virulent factor of soft tissue infections [4, 5]. In a large study published in 2015, the authors speculated that *S. pseudoporcinus* might not be associated with invasive disease to the same extent as *S. agalactiae* [3]. Nonetheless, since late 2019, six new cases of severe invasive *S. pseudoporcinus* infection have emerged in the literature: six cases of invasive *S. pseudoporcinus* infections with bacteremia (four cases of endocarditis, one associated with syphilis-human immunodeficiency virus, another with a multiloculated pleural empyema [11–14]), and an additional obstetric case of maternal sepsis and fetal demise [15]. Herein, we present a documented fatal case of SBP associated with bacteremia due to a multidrug-resistant *S. pseudoporcinus* strain that rapidly resulted in MODS. Risk factors associated with invasive infection are shown in Table 4, as they emerge collectively from the seven recently reported cases [11–15], including our case. *S. pseudoporcinus* could be an emerging multidrug-resistant pathogen, as it is now more easily recognizable with the new advanced biochemical techniques.

**Table 4:** Risk factors for *Streptococcus pseudoporcinus* acquisition and probable sites of colonization with subsequent bacteremia and severe invasive disease (6 cases [11–15] including the present case)

*Risk factors*	
Age	40–81 (mean 55) years
Diabetes mellitus	2/7
Hypertension	2/7
Immunosuppression	3/7
Chronic heart failure	3/7
Obesity	1/7
*Probable sites of Streptococcus pseudoporcinus* colonization	
Gastrointestinal tract/oropharynx	(6/7)
Genitourinary tract	(1/7)

The pathogenetic role of *S. pseudoporcinus* and its prevalence in humans warrants further investigation, and may be currently underestimated due to its misidentification as *S. agalactiae*. Recent studies underline the potential of *S. pseudoporcinus* to cause severe, invasive infections via gastrointestinal/oropharynx colonization and subsequent bacteremia, leading to life-threatening diseases [11–14]. New techniques for bacterial identification in routine clinical microbiology may affect its known prevalence, revealing its true significance as a human pathogen. *S. pseudoporcinus* resistance to penicillin, to third-generation cephalosporins, and even to carbapenems was reported in a previous case [5], and is consistent with the antibiotic susceptibility of our isolate. To date, multidrug-resistant *S. pseudoporcinus* has been found to be susceptible to vancomycin, daptomycin, linezolid, levofloxacin, clindamycin, and tetracycline. *S. pseudoporcinus* should be identified as early as possible in such cases, and administration of empirical antibiotics should provide wide coverage against common microorganisms as well as against this important, potentially life-threatening streptococcus.

## Data Availability

Not applicable.

## Abbreviations

BP: Blood pressure
bpm: Beats per minute
CDC: Centers for Disease Control and Prevention
CRP: C-Reactive protein
GBS: Group B *Streptococcus*
LDH: Lactate dehydrogenase
MIC: Minimum inhibitory concentration
MODS: Multiple-organ dysfunction syndrome
WBC: White blood cells
